# Disease and outcome disparities in multiple myeloma: exploring the role of race/ethnicity in the Cooperative Group clinical trials

**DOI:** 10.1038/s41408-018-0102-7

**Published:** 2018-07-06

**Authors:** Sikander Ailawadhi, Susanna Jacobus, Rachael Sexton, Alexander K. Stewart, Angela Dispenzieri, Mohamad A. Hussein, Jeffrey A. Zonder, John Crowley, Antje Hoering, Bart Barlogie, Robert Z. Orlowski, S. Vincent Rajkumar

**Affiliations:** 1 Mayo Clinic, Jacksonville, FL USA; 2 Dana Farber Cancer Institute–ECOG-ACRIN Biostatistics Center, Boston, MA USA; 3 South West Oncology Group (SWOG) Statistical Center, Seattle, WA USA; 4 Mayo Clinic, Scottsdale, AZ USA; 5 Mayo Clinic, Rochester, MN USA; 6 Celgene Corporation, Summit, NJ USA; 7 Karmanos Cancer Institute, Wayne State University, Detroit, MI USA; 8 Mount Sinai Medical Center, New York, NY USA; 9 MD Anderson Cancer Center, The University of Texas, Houston, TX USA

## Abstract

Multiple myeloma (MM) is an incurable hematologic malignancy with disparities in outcomes noted among racial-ethnic subgroups, likely due to disparities in access to effective treatment modalities. Clinical trials can provide access to evidence-based medicine but representation of minorities on therapeutic clinical trials has been dismal. We evaluated the impact of patient race-ethnicity in pooled data from nine large national cooperative group clinical trials in newly diagnosed MM. Among 2896 patients enrolled over more than two decades, only 18% were non-White and enrollment of minorities actually decreased in most recent years (2002–2011). African-Americans were younger and had more frequent poor-risk markers, including anemia and increased lactate dehydrogenase. Hispanics had the smallest proportion of patients on trials utilizing novel therapeutic agents. While adverse demographic (increased age) and clinical (performance status, stage, anemia, kidney dysfunction) factors were associated with inferior survival, patient race-ethnicity did not have an effect on objective response rates, progression-free, or overall survival. While there are significant disparities in MM incidence and outcomes among patients of different racial-ethnic groups, this disparity seems to be mitigated by access to appropriate therapeutic options, for example, as offered by clinical trials. Improved minority accrual in therapeutic clinical trials needs to be a priority.

## Introduction

Multiple myeloma (MM) is the second most common hematologic malignancy with approximately 30,000 new patients diagnosed in the United States (U.S.) every year^1^. MM incidence is noted to have increased over time with a progressively younger median age at diagnosis^2^. At the same time, owing to a rapidly improving therapeutic landscape, death rates have been falling over the past decade with a reported 5-year survival rate of 48.5%^1–4^. While these trends hold true for the U.S. population as a whole, with changing population demographics there is an increasing focus on how various population subgroups may be benefiting from survival improvement in MM. This is especially true for population subgroups by race and ethnicity, as several studies have shown that significant racial disparities exist in MM incidence and outcomes^1,3,5–7^. These disparities have been explored previously and a differential access to treatment as well as health-care utilization have been identified as a potential cause^5,8–10^. Such a finding has been especially true regarding access to stem cell transplant (SCT), a standard of care for MM treatment, which is known to be utilized much more commonly in Whites as compared to racial-ethnic minorities^5,10–12^. Nevertheless, some retrospective, single-institution and single clinical trial analyses have shown that, in equal access situations, racial disparities in outcomes may be mitigated, especially for those who receive SCT^12–14^. Such an analysis has not been done on a larger, national scale across multiple clinical trials and especially not in prospectively collected data for novel therapeutic agents irrespective of SCT. We analyzed patient-level data from published Eastern Cooperative Oncology Group (ECOG)-ACRIN/South West Oncology Group (SWOG) clinical trials in newly diagnosed MM (NDMM) patients to explore the interplay of race/ethnicity and outcomes.

## Methods

Data from nine previously published ECOG-ACRIN/SWOG clinical trials in NDMM between 1988 and 2011 was pooled for this analysis. The identified clinical trials included E1A06, E4A03, E1A00, E2A02, E5A93, E9486, S0204, S0232, and S9321. Pertinent details of the selected clinical trials are shown in Table 1. Data collected on the patients enrolled in these trials included baseline demographics (age, gender, race-ethnicity), clinical features (ECOG performance status, presence of lytic lesions, serum calcium, hemoglobin (Hb), creatinine, beta-2-microglobulin, lactate dehydrogenase (LDH), body mass index (BMI)), and MM characteristics (International Staging System (ISS) disease stage, heavy and light chain category, bone marrow plasma cell percentage). Clinical trials were grouped into fixed duration vs. treatment given to progression (including maintenance or not), with or without SCT, including a novel agent (proteasome inhibitor or immunomodulatory drug) or not, and the years of patient enrollment (1988–1991, 1992–2001, 2002–2011).

**Table 1:** Clinical trial details

Study	Phase	Intervention	*N* evaluable patients	% Total	Median follow-up (years)^a^	*N* survival events^b^
S9321	3	VBMCP vs. Mel+TBI+SCT	788	27.2	9.0	481
S0204	2	Thal→ Tandem SCT→Thalidomide maintenance	130	4.5	9.9	53
S0232	3	Dex+/−Len	176	6.1	6.7	89
E9486	3	VBMCP vs. VBMCP+IFN α vs. VBMCP+Cyclophosphamide	648	22.4	12.8	492
E5A93	3	VBMCP vs. VBMCP+IFN α or Cyclophosphamide	222	7.7	9.9	164
E1A00	3	Dex+/−Thal	205	7.1	8.0	128
E2A02	2	Bortezomib	40	1.4	6.0	25
E4A03	3	Len+low vs. high dose Dex	402	13.9	7.0	191
E1A06	3	Mel, Prednisone+Thal vs. Len	285	9.8	4.9	159

*VBMCP* Vincristine/BCNU/Melphalan/Cyclophosphamide/Prednisone, *Mel* myeloablative melphalan, *TBI* total body irradiation, *SCT* stem cell transplant, *Len* lenalidomide, *Thal* thalidomide, *Dex* dexamethasone, *IFN* interferon

^a^In the absence of censoring

^b^Follow-up was censored at 6 years for all studies

Patient were divided into mutually exclusive race-ethnicity categories, including non-Hispanic Whites (NHW), non-Hispanic African-Americans (NHAA), non-Hispanic Others (NHO), and Hispanics. Associations between race-ethnicity and categorical baseline patient, disease, and study design characteristics were assessed using the chi-square test while that between race-ethnicity and continuous baseline patient and disease characteristics were assessed using Wilcoxon test. Survival distributions since the time of enrollment were estimated based on the Kaplan–Meier method. Cox proportional hazards regression was used to estimate hazard ratios (HRs) in univariate and multivariable models. For multivariable models, covariate distribution was reviewed visually for proportional hazards. All patients were included so “missing” was included as a category, if appropriate. Wald test *p* values were provided from Cox regression models. Time to event data was censored at 6 years to have more uniform follow-up among the included studies. No adjustments were made for multiple testing. SAS 9.4 software was used for statistical analysis.

## Results

A total of 3026 patients were included in the analysis from the nine selected clinical trials. Of these, 3002 patients had data available on race. Furthermore, 106 patients had missing information on ethnicity, leading to the final study cohort of 2896 patients. Patient and clinical trial characteristics are shown in Table 2. Gender distribution comprised 1693 (58.5%) males and 1203 (41.5%) females. Racial-ethnic subgroups included 2373 (81.9%) NHW, 392 (13.5%) NHAA, 76 (2.6%) Hispanics, and 55 (1.9%) NHO. Median age for the whole cohort was 61.4 years (range: 23.2–91.9 years). ISS stage was missing in 469 patients. Of the rest, ISS stage was I–II in 71.4% patients and stage III in 28.6% patients. Lytic bony lesions were present in 63.9% patients. Looking at common laboratory parameters, majority of the patients (96.2%) did not have hypercalcemia (serum calcium >12 mg/dL) and 86.8% did not have any significant elevation in serum creatinine (creatinine ≥2 mg/dL). Significant anemia (Hb ≤ 10 g/dL) was noted in 39.4% patients. We explored BMI distribution among the patients and noted that, of the 1261 patients with BMI data available, 40.7% were classified as overweight (25 ≤ BMI < 30) and 25.4% as obese (BMI ≥ 30).

**Table 2:** Clinical trial and patient characteristics

	*N* = 2896	Percentage (%)
Clinical trial characteristic		
Enrollment year		
1988–1991	591	20.4
1992–2001	1057	36.5
2002–2011	1248	43.1
Novel agent^a^ utilized		
Yes	1087	37.5
No	1809	62.5
Stem cell transplant utilized		
Yes	918	31.7
No	1978	68.3
Duration of treatment		
Fixed duration	607	21.0
Treatment to progression	2289	79.0
Patient characteristic		
Race-ethnicity		
Non-Hispanic White	2373	81.9
Non-Hispanic African Americans	392	13.5
Non-Hispanic other	55	1.9
Hispanic	76	2.6
Age		
<70 years	2195	75.8
≥70 years	701	24.2
Gender		
Male	1693	58.5
Female	1203	41.5
International Staging System		
ISS I–II	1733	71.4
ISS III	694	28.6
Missing	469	
ECOG performance status		
PS = 0	1006	35.0
PS > 0	1869	65.0
Missing	21	
Hemoglobin		
Hb > 10 g/dL	1750	60.6
Hb ≤ 10 g/dL	1136	39.4
Missing	10	
Lytic lesions		
LL absent	1040	36.1
LL present	1838	63.9
Missing	18	
Calcium		
Ca ≤ 12 mg/dL	2759	96.2
Ca > 12 mg/dL	109	3.8
Missing	28	
Creatinine		
Cr < 2 mg/dL	2238	86.8
Cr ≥ 2 mg/dL	340	13.2
Missing	318	

^a^Novel agent = proteasome inhibitors or immunomodulatory drugs

### Patient characteristics by race

Median age was significantly different (*p* < 0.001) with NHO being the youngest (median 58.1 years, range 40.1–83.0) and NHW being the oldest (median 62 years, range 23.2–91.9). Gender distribution was also significantly different among patient race-ethnicity subgroups, with males comprising 67.1% of Hispanics, 59.6% of NHW, 51.8% of NHAA, but only 45.5% of NHO (*p* = 0.002). We noted that NHAA had the highest proportion of patients classified as obese (27.6%) and NHO had the lowest (12.9%), while NHW had the highest proportion of patients classified as overweight (41.4%) with NHO again having the lowest (29%) (*p* = 0.018). Among laboratory values evaluated, significant anemia (Hb ≤ 10 g/dL) was noted in 55.9% NHAA, 49.1% NHO, 44.0% Hispanics, but only in 36.3% NHW (*p* < 0.001). Similarly, LDH was distributed significantly differently among race-ethnicity subgroups, with NHAA having the highest mean LDH (270.9 U/L) and NHO having the lowest (207.5 U/L) (*p* = 0.011). There were no significant differences among patients for ECOG performance status, ISS stage, presence or absence of lytic bony lesions, bone marrow plasmacytosis, serum calcium, or creatinine level.

### Patient participation in the selected clinical trials by race-ethnicity

There were significant differences noted among patients of different racial-ethnic subgroups for all the clinical trial characteristics studied. We noted that, compared to 1988–1991, racial-ethnic minority enrollment in clinical trials increased in 1992–2001, with NHAA increasing from 9.5 to 16.5% and Hispanics increasing from 2.5 to 3.7%. There was a reciprocal decrease noted in NHW enrollment over the same time periods (85.6–78.3%). But in more recent years (2002–2011), minority enrollment has decreased again, with NHAA decreasing to 13% and Hispanics to 1.8% (*p* < 0.001). Majority of patients in all racial-ethnic subgroups were enrolled in clinical trials not involving a novel anti-MM agent. Despite this, NHW had the highest proportion of patients treated on novel therapeutic agent clinical trials (38.5%) while Hispanics had the smallest proportion (25%) (*p* = 0.047). Similarly, majority of patients for all racial-ethnic subgroups were enrolled on clinical trials not involving SCT, but there was a significant difference in the distribution with NHAA having the highest proportion of patients on SCT clinical trials (38%) and NHO the lowest (27.3%) (*p* = 0.034). With respect to fixed duration treatment vs. treatment to progression as well, there were significant differences with 22.1% of NHW but only 5.3% of Hispanics receiving treatment on trials with fixed duration of therapy (*p* = 0.001).

### Outcomes by patient characteristics other than race-ethnicity

All the collected demographic and clinical patient characteristics were evaluated for differences in survival in univariate and multivariable models. The study cohort follow-up was censored at 6 years, yielding 2375 progression events and 1782 deaths. Progression-free survival (PFS) was slightly better for females as compared to males (HR 0.92, 95% confidence interval (CI) 0.84, 0.99), while it was significantly worse for those with age ≥70 years (HR 1.17, 95% CI 1.05, 1.31) or an ECOG performance status >0 (HR 1.23, 95% CI 1.12, 1.34). Similarly, patients with ISS stage III (reference ISS stage I–II) had significantly worse PFS (HR 1.20, 95% CI 1.07, 1.34). Among laboratory parameters tested, PFS was significantly worse for patients with Hb ≤ 10 g/dL (HR 1.22, 95% CI 1.11, 1.33) and for those with serum creatinine ≥2 mg/dL (HR 1.19, 95% CI 1.03, 1.36). PFS was borderline significant for the presence of lytic lesion(s) (HR 1.08, 95% CI 0.99, 1.18) and for patients with serum calcium >12 mg/dL (HR 1.19, 95% CI 0.97, 1.47).

**Fig. 1:**
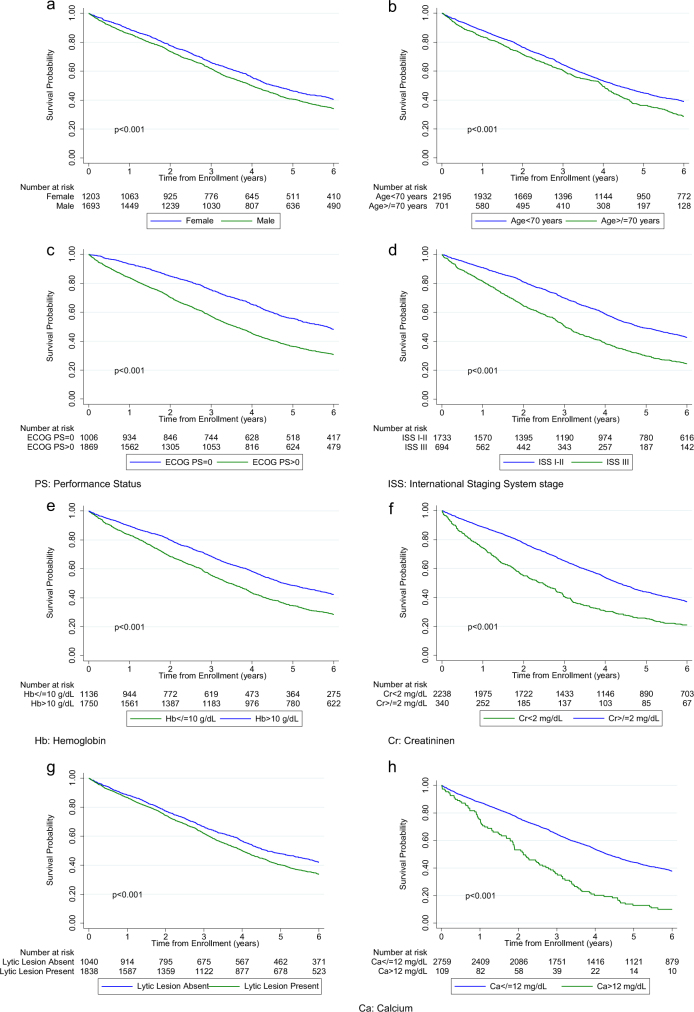
**Overall survival of patients included in the study, by various demographic and clinical myeloma-associated characteristics studied**. **a** Overall survival for all patients analyzed by gender. **b** Overall survival for all patients analyzed by patient age (≥70 or <70 years). **c** Overall survival for all patients analyzed by ECOG performance status (ECOG > 0 or ECOG = 0). **d** Overall survival for all patients analyzed by International Staging System; ISS Stage (ISS I–II or ISS III). **e** Overall survival for all patients analyzed by anemia (hemoglobin >10 or ≤10 g/dL). **f** Overall survival for all patients analyzed by kidney function (serum creatinine <2 or ≥2 mg/dL). **g** Overall survival for all patients analyzed by the presence of lytic lesions (presence or absence of lytic lesions). **h** Overall survival for all patients by the presence of hypercalcemia (serum calcium ≤12 mg/dL or >12 mg/dL)

All the same characteristics that were associated with PFS differences were associated with significant differences in overall survival (OS) as well, with a more pronounced effect. These included worse OS associated with male gender (HR for females 0.83, 95% CI 0.75, 0.91, Fig. 1a), age ≥70 years (HR 1.30, 95% CI 1.15, 1.48, Fig. 1b), ECOG performance status >0 (HR 1.46, 95% CI 1.31, 1.62, Fig. 1c), ISS stage III (HR 1.34, 95% CI 1.18, 1.52, Fig. 1d), Hb ≤ 10 g/dL (HR 1.35, 95% CI 1.22, 1.49, Fig. 1e), and serum creatinine ≥2 mg/dL (HR 1.21, 95% CI 1.03, 1.40, Fig. 1f). In addition, the presence of lytic lesions (HR 1.14, 95% CI 1.03, 1.26, Fig. 1g) and serum calcium >12 mg/dL (HR 1.50, 95% CI 1.20, 1.86, Fig. 1h) were also associated with a significant worsening of median OS.

### Outcomes by patient race-ethnicity

**Fig. 2:**
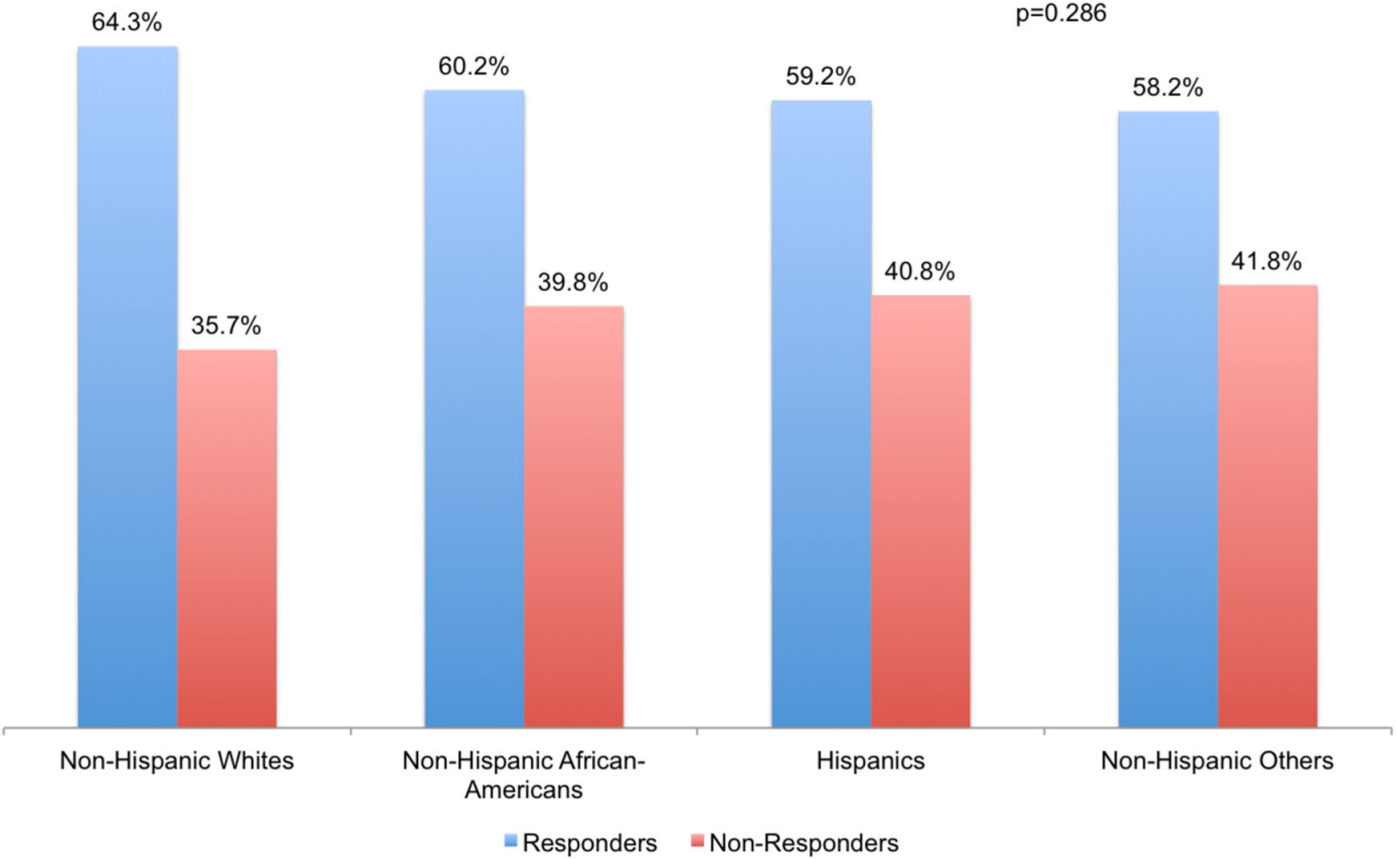
Comparison of response rates to the primary objectives of included clinical trials by patient race-ethnicity

**Fig. 3:**
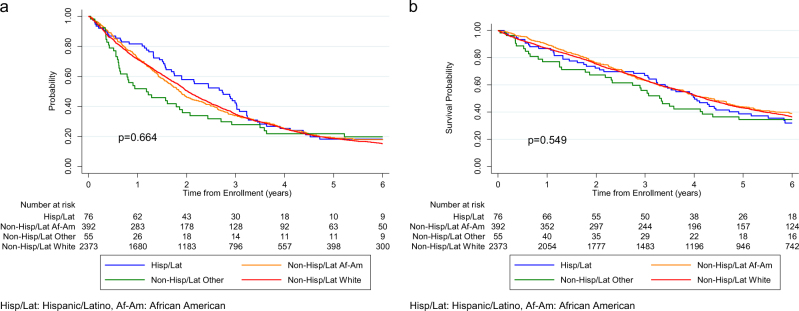
**Survival outcomes for patients included in the study by race-ethnicity**. **a** Progression-free survival (PFS) by patient race-ethnicity. **b** Overall survival (OS) by patient race-ethnicity

Response rates for the primary objectives of clinical trials included were compared among patients of different race-ethnicity groups. There was no statistically significant difference noted for this efficacy analysis (*p* = 0.286) (Fig. 2). Median PFS for the whole study cohort was 2.0 years while the median OS was 4.2 years. Median PFS and OS by race-ethnicity categories are shown in Table 3. Using Hispanics as the reference in the multivariable model, PFS was not noted to be significantly different for any of the race-ethnicity groups (*p* = 0.427) including NHW (HR 1.20, 95% CI 0.92, 1.55), NHAA (HR 1.14, 95% CI 0.86, 1.51), and NHO (HR 1.32, 95% CI 0.88, 1.96) (Fig. 3a). Similarly, OS was not noted to be significantly different (*p* = 0.760) for NHW (HR 1.00, 95% CI 0.75, 1.33), NHAA (HR 0.95, 95% CI 0.70, 1.29), or NHO (HR 1.13, 95% CI 0.73, 1.75) with Hispanics again being the reference (Fig. 3b).

**Table 3:** Progression-free and overall survival by patient race-ethnicity

Race-ethnicity	Progression-free survival		Overall survival	
	*N* (events/patients)	Median (years) (95% CI)^a^	*N* (events/patients)	Median (years) (95% CI)^a^
Non-Hispanic White	1960/2373	2.0 (1.9, 2.1)	1466/2373	4.2 (4.0, 4.4)
Non-Hispanic African-American	314/392	1.9 (1.7, 2.1)	232/392	4.3 (3.8, 4.8)
Hispanic	60/76	2.7 (1.7, 3.0)	50/76	4.0 (3.2, 4.9)
Non-Hispanic other	41/55	1.2 (0.6, 1.9)	34/55	3.3 (2.3, 4.4)

*CI* confidence interval

## Discussion

Outcomes in MM have improved significantly over the past two decades^2,4,10^. Increasing survivorship has led to focus on how the therapeutic advancements may have benefited various subgroups of MM patients. An important area that deserves focus is patient race-ethnicity. Historically, there has been some work done to understand disease incidence, progression, outcomes, and, to some extent, biologic differences between Caucasians (Whites) and AA, the two largest racial subgroups in the U.S. population^6,7,13,14^. With changing demographic landscape, such an analysis needs to be broadened to include especially Hispanics, the fastest growing racial-ethnic subgroup in the country^15^. Existing data suggests that outcomes among MM patients of different racial-ethnic groups are different and these trends are changing over time^3,5,10^. Causes for this can indeed be multifactorial, but most of the currently available research suggests that access to various therapeutic options may be most important^9,10,12^. Clinical trials present a unique opportunity for addressing this question as they contain carefully collected, clinically annotated data, best equipped to perform secondary analyses. The current undertaking involving nine large, national clinical trials in MM conducted at the Cooperative Group level is well positioned for such an analysis.

A higher proportion of patients in our analysis were enrolled in recent years, suggesting increased clinical trial enrollment overall. But we noted a very small proportion of patients in the cohort belonging to racial-ethnic minorities, consistent with other reports of clinical trial enrollment nationally^16–18^. Similar to a previous report showing increased enrollment of AA patients in clinical trials over time^17^, we noted that, compared to 1988–1991, the number of AA and Hispanics enrolled in clinical trials improved in years 1992–2001. But we also noticed that, in more recent years (2002–2011), this trend actually reversed, and at least in the MM Cooperative Group clinical trials, the enrollment of racial-ethnic minorities decreased. This is contrary to the increasing proportion of racial-ethnic minorities in the U.S. population, representing approximately 30% of MM patients in the U.S. currently^2,15^. This is a concerning trend despite a requirement by the National Institute of Health that members of minority populations be represented adequately in clinical research^19^. A possible contributor to decreased minority enrollment in the Cooperative Group trials may be their enrollment in other national or regional clinical trials, e.g., those run by pharmaceutical companies or academic institutions. So far, trends of increased minority enrollment in these other settings have not been reported to corroborate this potential hypothesis. Considering that racial minorities may bear the largest burden of cancer in the U.S.^20^, their under-representation in clinical trials is a huge source of preventable health-care disparity that needs to be addressed urgently.

We observed that NHW were the ones more commonly enrolled on trials with novel therapeutic agents while Hispanics were least likely to go on to such trials. This could be due to practice patterns, differences in access to certain trials across the country, and even differences in insurance coverage, which is responsible for the non-investigational aspects of a clinical trial. We noted that majority of patients did not get a SCT-based clinical trial, although among those who did, NHAA had the highest proportion of patients. This may again be reflective of differences in practice patterns and geographical availability of trials, since it is known that, overall, racial-ethnic minorities are much less likely to receive SCT for MM^10–12^.

Racial-ethnic minorities (NHAA and Hispanics) had a younger median age than NHW in our analysis, which has significant implications on outcomes, as age has been noted as an independent prognostic factor in MM^3^. The gender distribution differences are not necessarily representative of disease incidence by age but may suggest access to and enrollment into clinical trials. Among Hispanics, males were more commonly enrolled as compared to NHAA and NHW. These trends may be helpful in addressing clinical trial enrollment in the future and making sure that access is uniform among demographic subgroups including gender among race-ethnicities. Obesity has been noted as an independent risk factor for MM in several large analyses^21^. It has been reported that obesity is a more prevalent problem in racial-ethnic minorities in the U.S., thus possibly putting them at a higher risk of developing MM^22,23^. While we noted a higher incidence of obesity among the NHAA in our cohort, patient BMI controlling for race-ethnic categories was not associated with differences in PFS or OS. It seems access to effective therapy with clinical trial participation overcame any potential effects of obesity on outcomes. To our knowledge, no prior analysis has looked at patient outcomes in MM by obesity rates and race.

We report significant differences in certain laboratory parameters associated with more aggressive disease, including higher incidence of significant anemia and LDH in NHAA. Other reports have also shown a higher incidence of certain myeloma-defining events, including anemia, in NHAA^10^. Furthermore, in our analysis clinical and demographic parameters, including advanced age, higher disease stage, poor performance status, presence of lytic bony lesions, significant anemia, hypercalcemia and renal dysfunction, were associated with worse outcomes, similar to prior reports^24^. Our observation is important, as, despite adverse prognostic markers in certain racial-ethnic groups, patients had similar response rates and survival, underscoring the importance of access to appropriate therapeutic options, e.g., participation in clinical trials. To this end, we report that there were no differences in objective response rates, PFS, or OS by race-ethnicity across the nine included national clinical trials. While there was heterogeneity in the clinical trials included, the proportion of minority enrollment was similar across various trials, rendering validity to this unique observation. This is in accordance with smaller previous reports that have evaluated single-institution or single-trial data^13,14^. We confirm these findings on a larger scale spanning many more years of patient care.

We report the analysis from a large, prospectively collected national data, which shows low clinical trial accruals for racial-ethnic minorities. Nevertheless, despite heterogeneity in patient characteristics including some poor-risk features, patients from racial-ethnic minorities had similar outcomes to NHW, the most numerous group. This underscores the therapeutic benefit that evidence-based and novel treatment options, typically offered in prevalent clinical trials, bring to patients. Ongoing efforts regarding improving clinical trial accrual in the U.S., especially among racial-ethnic minorities, need to be strengthened further, which may require a concerted focus from patients, health-care and advocacy groups as well as political will.

## References

[1] Siegel RL, Miller KD, Jemal A (2017). Cancer Statistics, 2017. CA Cancer J. Clin..

[2] Costa LJ (2017). Recent trends in multiple myeloma incidence and survival by age, race, and ethnicity in the United States. Blood Adv..

[3] Ailawadhi S (2012). Outcome disparities in multiple myeloma: a SEER-based comparative analysis of ethnic subgroups. Br. J. Haematol..

[4] Kumar SK (2014). Continued improvement in survival in multiple myeloma: changes in early mortality and outcomes in older patients. Leukemia.

[5] Ailawadhi S, Bhatia K, Aulakh S, Meghji Z, Chanan-Khan A (2017). Equal treatment and outcomes for everyone with multiple myeloma: are we there yet?. Curr. Hematol. Malig. Rep..

[6] Gebregziabher M, Bernstein L, Wang Y, Cozen W (2006). Risk patterns of multiple myeloma in Los Angeles County, 1972-1999 (United States). Cancer Causes Control.

[7] Waxman AJ (2010). Racial disparities in incidence and outcome in multiple myeloma: a population-based study. Blood.

[8] Ailawadhi S (2016). Impact of access to NCI- and NCCN-designated cancer centers on outcomes for multiple myeloma patients: a SEER registry analysis. Cancer.

[9] Ailawadhi S (2017). Racial disparity in utilization of therapeutic modalities among multiple myeloma patients: a SEER-medicare analysis. Cancer Med..

[10] Ailawadhi S., et al. Trends in multiple myeloma presentation, management, cost of care, and outcomes in the Medicare population: a comprehensive look at racial disparities. *Cancer* **124**, 1710-1721 (2018). 10.1002/cncr.31237 29360160

[11] Al-Hamadani M, Hashmi SK, Go RS (2014). Use of autologous hematopoietic cell transplantation as initial therapy in multiple myeloma and the impact of socio-geo-demographic factors in the era of novel agents. Am. J. Hematol..

[12] Schriber JR (2017). Hispanics have the lowest stem cell transplant utilization rate for autologous hematopoietic cell transplantation for multiple myeloma in the United States: a CIBMTR report. Cancer.

[13] Modiano MR, Villar-Werstler P, Crowley J, Salmon SE (1996). Evaluation of race as a prognostic factor in multiple myeloma. An ancillary of Southwest Oncology Group Study 8229. J. Clin. Oncol..

[14] Verma PS, Howard RS, Weiss BM (2008). The impact of race on outcomes of autologous transplantation in patients with multiple myeloma. Am. J. Hematol..

[15] *Annual Estimates of the Resident Population by Sex, Race, and Hispanic Origin for the United States: April 1, 2000 to July 1, 2009 (NC-EST2009-03)* (U.S. Census Bureau, Washington, D.C., 2010).

[16] Al-Refaie WB (2011). Cancer trials versus the real world in the United States. Ann. Surg..

[17] Kwiatkowski K, Coe K, Bailar JC, Swanson GM (2013). Inclusion of minorities and women in cancer clinical trials, a decade later: have we improved?. Cancer.

[18] Stewart JH, Bertoni AG, Staten JL, Levine EA, Gross CP (2007). Participation in surgical oncology clinical trials: gender-, race/ethnicity-, and age-based disparities. Ann. Surg. Oncol..

[19] Freedman LS (1995). Inclusion of women and minorities in clinical trials and the NIH Revitalization Act of 1993–the perspective of NIH clinical trialists. Control. Clin. Trials.

[20] Siegel R, Ward E, Brawley O, Jemal A (2011). Cancer statistics, 2011: the impact of eliminating socioeconomic and racial disparities on premature cancer deaths. CA Cancer J. Clin..

[21] Wallin A, Larsson SC (2011). Body mass index and risk of multiple myeloma: a meta-analysis of prospective studies. Eur. J. Cancer.

[22] *Obesity and Hispanic Americans* (U.S. Department of Health and Human Services: Rockville, MD, 2016).

[23] *Obesity and African Americans* (U.S. Department of Health and Human Services: Rockville, MD, 2016).

[24] Hanbali A, Hassanein M, Rasheed W, Aljurf M, Alsharif F (2017). The evolution of prognostic factors in multiple myeloma. Adv. Hematol..

